# Supplementary material for: OsMADS1 Regulates Grain Quality, Gene Expressions, and Regulatory Networks of Starch and Storage Protein Metabolisms in Rice
Source: Int J Mol Sci. 2023 Apr 28;24(9):8017. doi: 10.3390/ijms24098017 (PMC10178960; doi:10.3390/ijms24098017)
Supplement: Supplementary file 1 [file ijms-24-08017-s001.zip › ijms-2277424 Supplementary Materials.pdf]

International Journal of *Molecular Sciences*

Supplementary Materials

OsMADS1 Regulates Grain Quality, Gene Expressions, and Regulatory Networks of Starch and Storage Protein Metabolisms in Rice

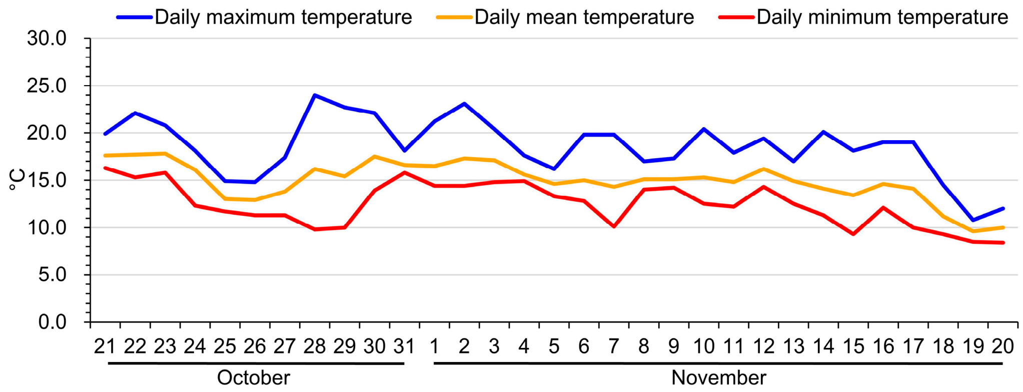

Figure S1. Analysis of daily temperatures during the flowering, grain filling and grain development stages of p*Ubi*::*OsMADS1* transgenic plants. Daily temperatures from Oct. 21th to Nov. 20th during the flowering, grain filling and grain development stages of control and T_0 transgenic plants of p*Ubi*::*OsMADS1* in Chengdu, Sichuan, China, 2019 are shown. The data of daily temperatures was provided by the Shuangliu district meteorological bureau of Chengdu, Sichuan, China.

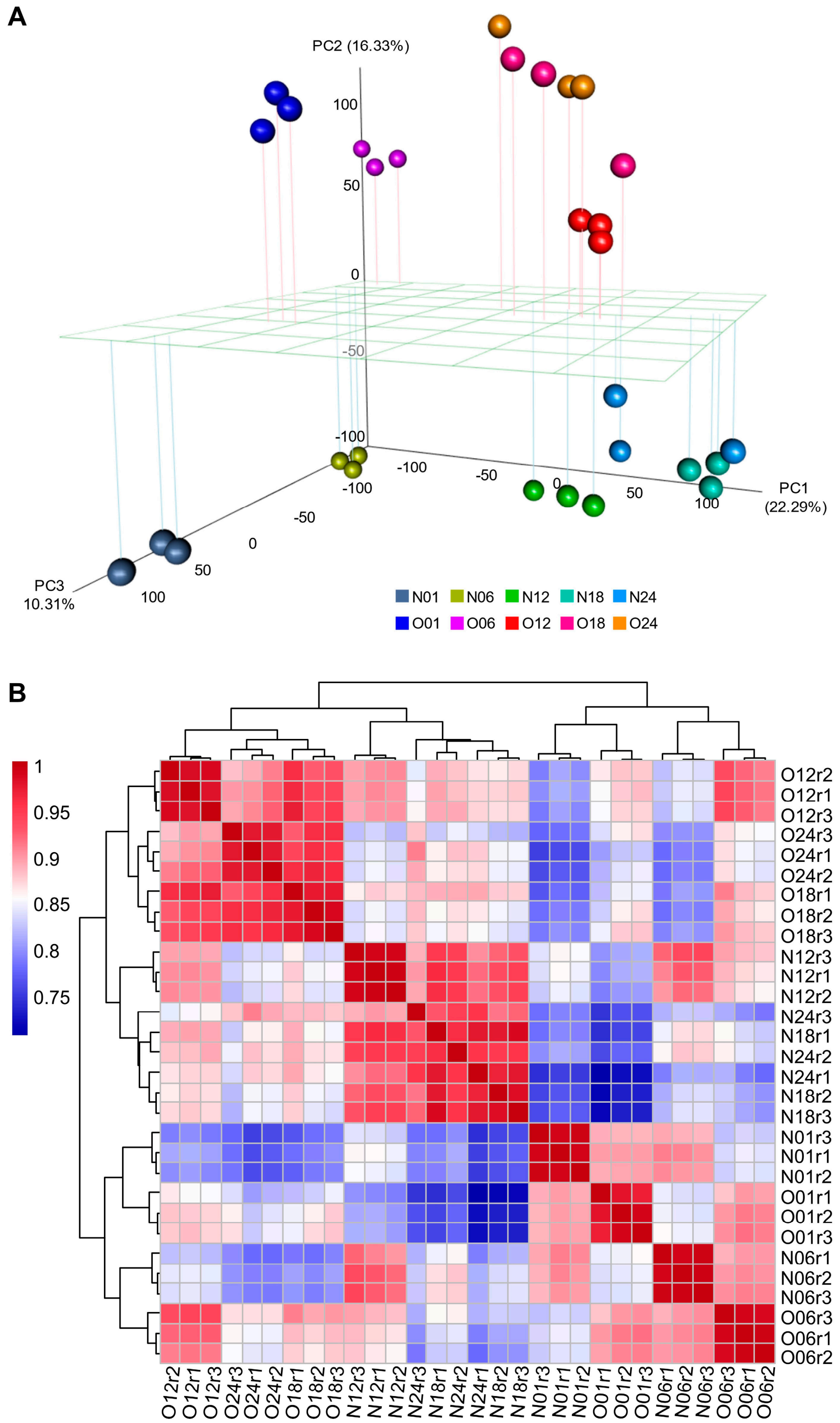

Figure S2. The principal component analysis (PCA) and sample-to-sample correlation analysis of grain samples from Nip and Olr. (**A**) PCA of grain samples from Nip and Olr. The closer distance between samples, the higher similarity of samples. (**B**) Cluster dendrogram of grain samples from Nip and Olr generated by sample-to-sample correlation analysis. The horizontal and vertical coordinates represent samples. The color of each cell located at the intersection of the two corresponding samples in the horizontal and vertical directions represents the Pearson's correlation coefficient calculated from the FPKM values of genes in the two samples. Color scale is shown at the upper left of the cluster dendrogram. The darker red of a cell, the higher correlation value of the cell, and the higher correlation and similarity between the two corresponding samples. N01, N06, N12, N18 and N24 represent 1 DAF, 6 DAF, 12 DAF, 18 DAF and 24 DAF grains of Nip, respectively; O01, O06, O12, O18 and O24 represent 1 DAF, 6 DAF, 12 DAF, 18 DAF and 24 DAF grains of Olr, respectively; N: Nip; O: Olr; DAF: Days After Fertilization (**A**,**B**). (**A**) The samples from Nip and Olr were clustered into two separated subgroups (Nip subgroup and Olr subgroup) in. (**A**,**B**) The samples from both Nip subgroup and Olr subgroup, the three biological replicates were tightly related at each time point and showed good correlation and similarity in general.

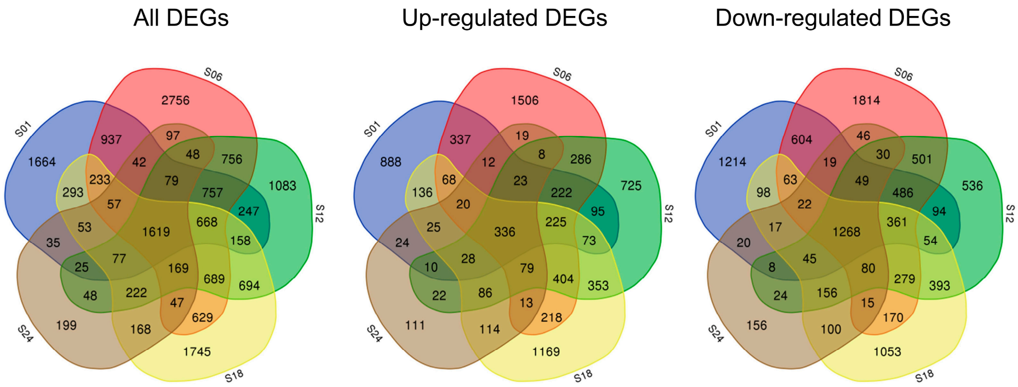

Figure S3. Analysis of DEGs in Nip and Olr during seed development by the Venn diagram. Venn diagram of DEGs between Nip and Olr at same time points. S01, S06, S12, S18 and S24 denote the DEGs sets obtained by comparing the grain samples at 1 DAF, 6 DAF, 12 DAF, 18 DAF and 24 DAF between Nip and Olr, respectively. Numbers in the figures represent the DEGs numbers. DAF: Days After Fertilization; DEGs: Differentially Expressed Genes; N: Nip; O: Olr; p-adjust < 0.01. In the comparison groups between Nip and Olr, 16294 unique DEGs (7635 up-regulated and 9775 down-regulated) and 1619 common DEGs (336 up-regulated and 1268 down-regulated) were identified. And 1664 (888 up-regulated and 1214 down-regulated), 2756 (1506 up-regulated and 1814 down-regulated), 1083 (725 up-regulated and 536 down-regulated), 1745 (1169 up-regulated and 1053 down-regulated) and 199 (111 up-regulated and 156 down-regulated) specific DEGs were found in NS01_vs_OS01, NS06_vs_OS06, NS12_vs_OS12, NS18_vs_OS18 and NS24_vs_OS24 datasets, respectively. In both unique DEGs and common DEGs of the five comparison groups, there are much less up-regulated DEGs than down-regulated DEGs. In addition, there are more up-regulated specific DEGs than down-regulated specific DEGs in NS12_vs_OS12 and NS18_vs_OS18 datasets, but less up-regulated specific DEGs than down-regulated specific DEGs in NS01_vs_OS01, NS06_vs_OS06 and NS24_vs_OS24 datasets.

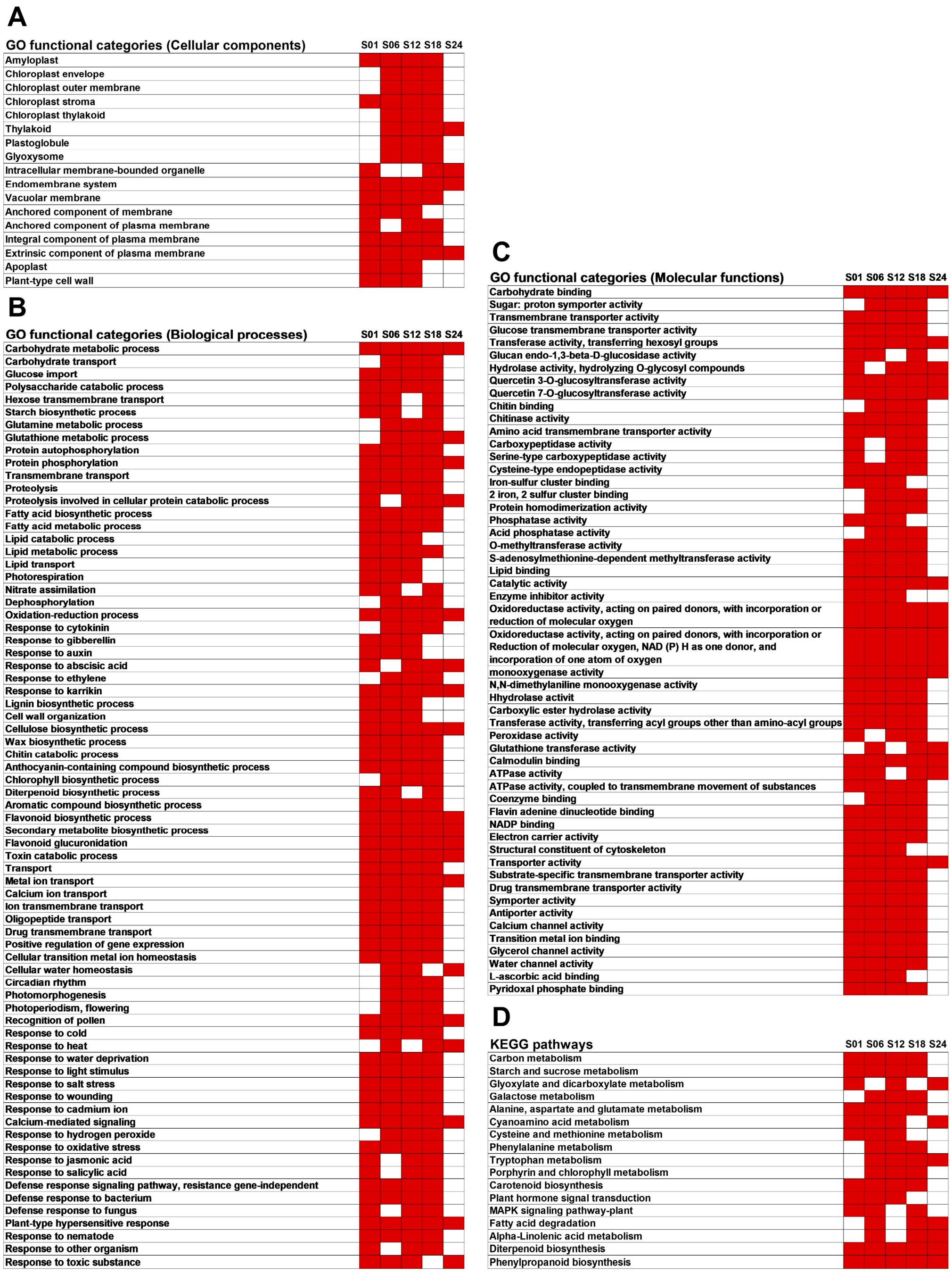

Figure S4. Enrichment analysis of DEGs between Olr and Nip grains. (**A-C**) GO enrichment [Cellular components (GO CC) (**A**), Biological processes (GO BP) (**B**) and Molecular functions (GO MF) (**C**)] analyses of DEGs between Olr and Nip grains. (**D**) KEGG enrichment of DEGs between Olr and Nip grains. Both GO terms and KEGG pathways were selected when they were enriched at least in three or more time points during grain development. Red bar represents the enriched GO terms (**A-C**) or KEGG pathways (**B**). DEGs: Differentially Expressed Genes; GO: Gene Ontology; KEGG: Kyoto Encyclopedia of Genes and Genomes; S01, S06, S12, S18 and S24 indicate grain samples at 1 DAF, 6 DAF, 12 DAF, 18 DAF and 24 DAF, respectively; DAF: days after fertilization; *p*-value < 0.05 (**A-D**). (**A-C**) With the p-value < 0.05 as the cutoff and based on enrichment at least in three of the five time points of grain samples for screening the enriched GO functional categories, 17, 73 and 53 enriched terms from GO CC, GO BP and GO MF functional categories were identified, respectively. (**A**) In the significantly enriched 17 GO CC terms, there are 15 terms involved in amyloplast, chloroplast components, organelles and components of endomembrane and cell membrane system including endoplasmic reticulum, Golgi apparatus, vacuoles, vesicles, as well as apoplast and cell wall. (**B**) Within the identified 73 GO BP terms, the biological processes involved in carbohydrate (such as glucose and hexose) transport, synthesis and metabolism, protein modification, transport and metabolism, basic biochemical and biological processes including carbon fixation, nitrate assimilation and oxidation-reduction process, as well as response to phytohormones like cytokinin, gibberellin and auxin which regulating grain development were significant enriched. (**C**) On the other hand, the GO MF terms related to the biochemical reactions and molecular functions including carbohydrate and sugar binding, transport, synthesis and metabolism, amino acid transport, protein modification such as disulfur bond isomerization, phosphorylation and methylation, protein hydrolysal metabolism, basic biochemical reactions of carbon and nitrogen compounds like catalysis, oxidation, reduction and hydrolysis, basic energy metabolism, as well as transport of sugar, protein and metal ions were significantly enriched in the identified 53 GO MF terms. (**D**) To further investigate functional distribution of the DEGs involved and enriched in various metabolic pathways, the KEGG pathway-based analysis was carried out; Among the enriched 17 pathways, DEGs were mostly enriched in the 12 pathways, including

carbon metabolism, carbohydrate metabolism such as starch and sucrose metabolism, amino acid metabolism associated with protein synthesis and metabolism, chlorophyll metabolism, and plant hormone signal transduction involved in regulation of grain development.

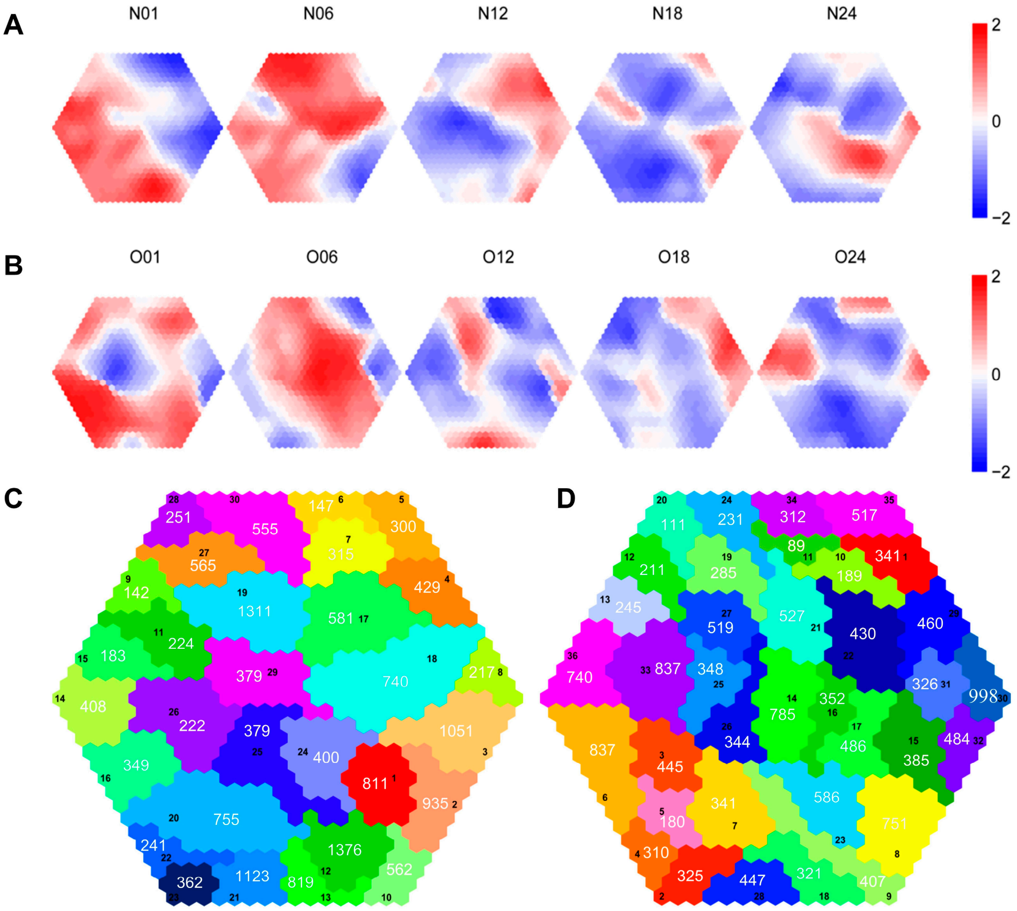

Figure S5. Analysis of temporal expression patterns of DEGs between Nip and Olr grains. (**A,B**) Self-organizing maps (SOMs) of DEGs between Nip (**A**) and Olr (**B**) grains at five representative time points during seed development. Each hexagon shows the component plane of the SOM of gene expression patterns at 1 DAF, 6 DAF, 12 DAF, 18 DAF and 24DAF grains of Nip and Olr, by using a color gradient from red to blue to indicate relatively higher and lower gene expression levels (see the colored bar). The smallest grid within each hexagon indicates a gene set which has similar expression patterns. N01, N06, N12, N18 and N24 represent 1 DAF, 6 DAF, 12 DAF, 18 DAF and 24 DAF grains of Nip, respectively; O01, O06, O12, O18 and O24 represent 1 DAF, 6 DAF, 12 DAF, 18 DAF and 24 DAF grains of Olr, respectively; N: Nip, O: Olr; DAP: Days After Fertilization; DEGs: Differentially Expressed Genes; *p*-adjust < 0.01 (**A,B**). (**C,D**) Robust clustering maps of DEGs of Nip (**C**) and Olr (**D**) grains during seed development. The irregular regions marked with different colors indicate different robust clusters, and genes in each cluster has the similar expression patterns of the five representative time points during seed development. The hexagonal lattices within each robust cluster means sub-clusters of genes with more similar expression patterns. And the black number and white number in each robust cluster represent the serial number and the total gene numbers of the corresponding robust cluster, respectively. (**A,B**) A matrix of expression values for the total DEGs between Nip and Olr was used as input data in the SOM algorithm, SOMs of Nip-group and Olr-group at the 1 DAF, 6 DAF, 12 DAF, 18 DAF and 24 DAF grains were constructed. Different gene sets with different expression patterns were self-organized into different smallest grids in the hexagonal SOMs, and genes within the same grid, adjacent grids and regions have similar expression patterns in. The DEGs in both Olr and Nip have relatively higher expression levels in 1 DAF and 6 DAF grains than 12 DAF, 18 DAF and 24DAF grains in general, which indicates more active gene expression in the early developmental stage of grains. However, the visualized temporal expression patterns between Nip-group and Olr-group are obviously different, indicating by specific expression levels among certain adjacent grids and regions in the SOMs

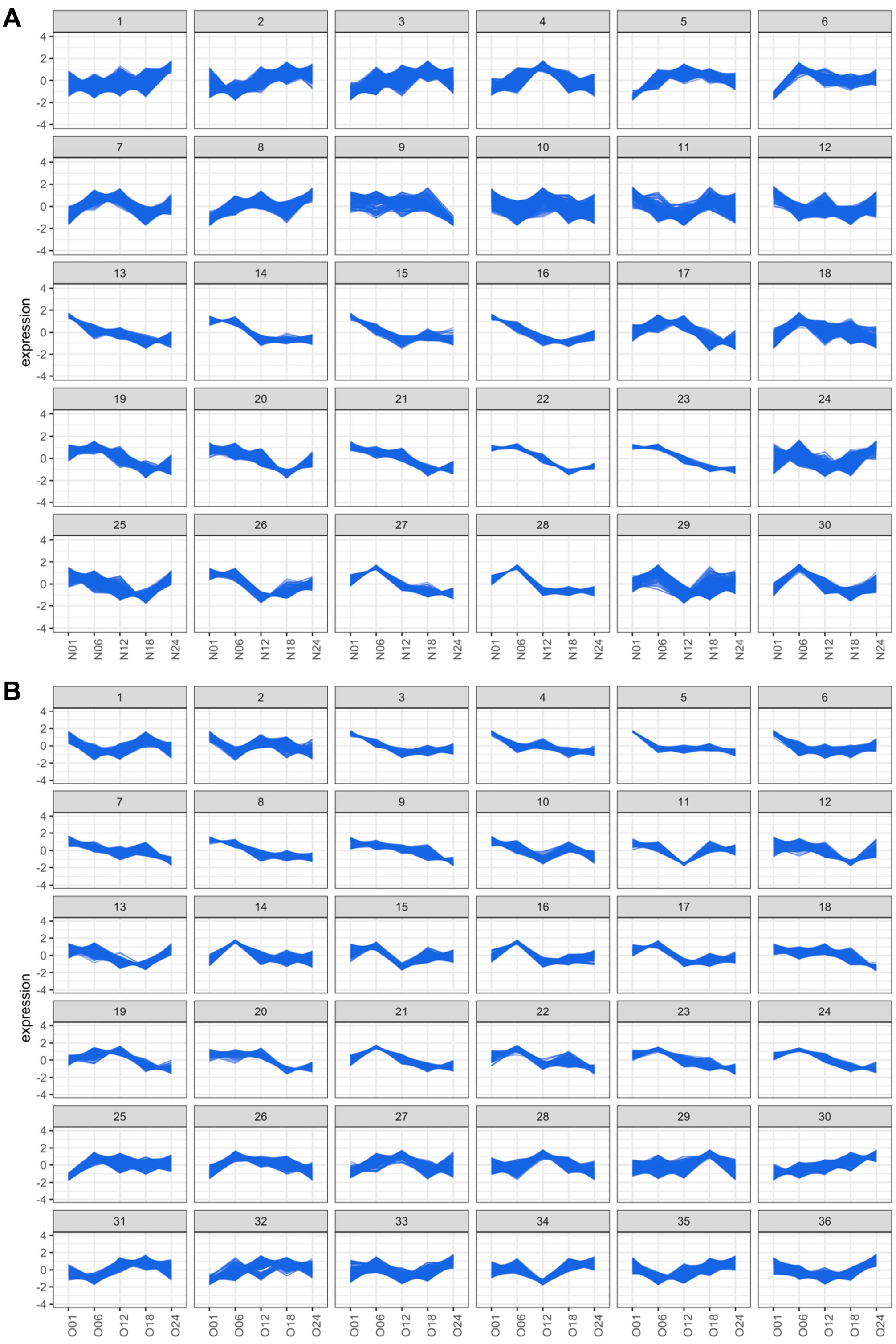

Figure S6. Analysis of temporal expression profiles for the robust clusters of DEGs between Nip and Olr grains. The insets show temporal expression profiles of thirty and thirty-six robust clusters in Nip (**A**) and Olr (**B**), respectively. The numbers besides the Y-axis represent the homogenized gene expression values; and homogenization of gene expression values is based on the expression levels of all the DEGs between Nip and Olr at all the five representative time points (1 DAF, 6 DAF, 12 DAF, 18 DAF and 24DAF) during seed development. N01, N06, N12, N18 and N24 represent 1 DAF, 6 DAF, 12DAF, 18DAF and 24 DAF grains of Nip, respectively; O01, O06, O12, O18 and O24 represent 1 DAF, 6 DAF, 12DAF, 18DAF and 24 DAF grains of Olr, respectively. N: Nip; O: Olr. DAF: days after fertilization. DEGs: Differentially Expressed Genes.

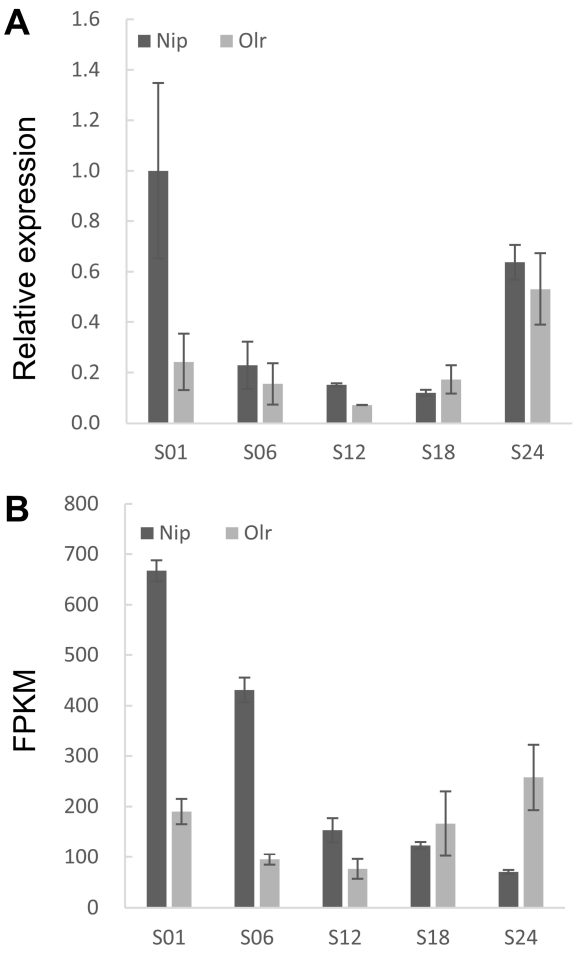

Figure S7. Validation of the mRNA-seq data by comparing the expression patterns of *OsMADS1* / *OsMADS1*^{Olr} through qRT-PCR and FPKM dada. (**A**,**B**) The relative expression levels of *OsMADS1* and *OsMADS1*^{Olr} in grains at representative developmental stages in Nip and Olr respectively by qRT-PCR (**A**) and RNA-seq (**B**) analysis, respectively. The value of *OsActin* mRNA was used as an internal control for data normalization, and the expression levels of *OsMADS1* in NIP at S01 were set as 1.0 (**A**). FPKM: Fragments per kilobase of exon per million reads mapped (**B**). S01: grains at 1DAF; S06: grains at 6 DAF; S12: grains at 12 DAF; S18: grains at 18 DAF; S24: grains at 24 DAF. DAF: Days After Fertilization. Data presented are mean values ± SDs of three replicates.

Table S1. PCR primers used to identify T_0 positive transgenic plants of p*Ubi*::*OsMADS1* vector

Primer pair	Forward primer (5′ - 3′)	Reverse primer (5′ - 3′)	Product size amplified from *OsMADS1*[Olr] genomic DNA (gDNA) and *OsMADS1* cDNA, respectively (bp)
gc	AACCAAGCAC-TGCTTGATCAG	CTTTTTCCTCAAG TCTTTGTTGAG	174 (Amplified from *OsMADS1*[Olr] gDNA), 84 (Amplified from *OsMADS1* cDNA)

Table S2. Candidate gene analysis for the Olr grain quality phenotype

Chromosome	Position (bp)	Reference base (Nip)[a]	Altered base in maternal plant, Olr[b]	Base in paternal plant, Nip[c]	SNP-index (MO-bulk)[d]	SNP-index (PN-bulk)[e]	SNP-index (O-bulk)[f]	SNP-index (N-bulk)[g]	Δ(SNP-index) (O-bulk - N-bulk)[h]	Gene accession number	Gene position (bp)	Annotation of mutation[i]	Gene annotation
3	6008076	A	T	A	0.97	0.00	1.00	0.37	0.63	*Os03g0214900*	6006751-6011719	E3 (4); c.2529A>T; p.Glu843Asp; missense	Conserved hypothetical protein
3	6011138	T	C	T	1.00	0.00	1.00	0.39	0.61	*Os03g0214900*	6006751-6011719	E1 (4); c.4T>C; p.Tyr2His; missense	Conserved hypothetical protein
3	6061195	G	A	G	0.86	0.00	1.00	0.37	0.63	*Os03g0215400*	6052902-6061365	E1 (8); c.80G>A; p.Gly27Asp; missense	MADS-domain-containing protein, MADS-box transcription factor 1 (OsMADS1), sexual reproduction
3	6546162	G	T	G	1.00	0.00	1.00	0.38	0.62	*Os03g0225100*	6545153-6546478	E1 (1); c.253G>T; p.Ala85Ser; missense	Mitogen-activated protein kinase

[a] Nip represents the reference *japonica* cv. Nip (Nipponbare); [b] Olr represents the maternal plant, Olr (Oat-like rice) mutant; [c] Nip here represents the paternal plant, Nipponbare; [d] MO-bulk represents the bulk DNA sample extracted from nine individual maternal plants, Olr; [e] PN-bulk represents the bulk DNA sample extracted from nine individual paternal plants, Nip; [f] O-bulk represents the bulk DNA sample extracted from 152 F_2 (Olr × Nip) individual plants showing Olr phenotypes; [g] N-bulk represents the bulk DNA sample extracted from 152 F_2 (Olr ×NIP) individual plants showing normal phenotypes; [h] Δ(SNP-index) (O-bulk - N-bulk) means that SNP-index of O-bulk minus SNP-index of N-bulk; [i] for example, 'E3 (4); c.2529A>T; p.Glu843Asp; Missense' means that the nucleotide mutation occurred in the third exon of the *Os03g0214900* gene, which consists of four exons [E3 (4)]; the 2529th nucleotide base of the coding sequence (CDS) mutated from 'A' to 'T' (c.2529A>T), which resulted in a corresponding amino acid substitution from 'Glu' to 'Asp' at the 843th amino acid of the encoding protein (p.Glu843Asp); the mutation type of this amino acid substitution is a missense mutation (missense).

Table S3. Statistics of Illumina sequencing data

Sample name	Obtained reads	Obtained bases (Gbp)	Q20 base ratio (%)	Q30 base ratio (%)	GC content (%)
N01r1	49,320,338	7,398,050,700	96.19	90.95	58.52
N01r2	48,806,338	7,320,950,700	95.51	89.41	58.84
N01r3	49,352,486	7,402,872,900	95.25	88.99	57.90
N06r1	55,477,898	8,321,684,700	95.86	90.33	55.90
N06r2	44,609,400	6,691,410,000	96.12	90.80	55.99
N06r3	47,900,812	7,185,121,800	96.19	90.95	55.60
N12r1	87,893,220	13,183,983,000	95.52	89.79	56.61
N12r2	53,477,048	8,021,557,200	96.12	90.81	57.29
N12r3	45,014,822	6,752,223,300	96.15	90.90	56.78
N18r1	40,864,268	6,129,640,200	96.52	91.30	54.92
N18r2	38,519,056	5,777,858,400	96.83	91.94	52.44
N18r3	46,265,780	6,939,867,000	96.55	91.49	55.02
N24r1	48,670,928	7,300,639,200	96.82	91.91	54.72
N24r2	42,225,232	6,333,784,800	96.30	91.04	55.90
N24r3	48,717,822	7,307,673,300	95.91	90.23	59.15
O01r1	48,439,724	7,265,958,600	95.33	89.11	58.19
O01r2	50,105,488	7,515,823,200	95.28	89.00	58.66
O01r3	42,994,878	6,449,231,700	95.57	89.54	58.97
O06r1	48,604,524	7,290,678,600	95.43	89.35	55.88
O06r2	51,248,574	7,687,286,100	96.16	90.89	55.34
O06r3	45,656,886	6,848,532,900	96.52	91.56	55.46
O12r1	45,248,340	6,787,251,000	96.31	91.18	55.94
O12r2	47,401,148	7,110,172,200	96.48	91.54	56.95
O12r3	51,264,334	7,689,650,100	96.33	91.19	56.78
O18r1	53,386,888	8,008,033,200	96.72	91.78	55.84
O18r2	46,613,432	6,992,014,800	95.96	90.35	57.47
O18r3	43,414,356	6,512,153,400	96.13	90.67	57.17
O24r1	48,092,878	7,213,931,700	95.92	90.35	57.74
O24r2	64,320,890	9,648,133,500	96.09	90.58	56.60
O24r3	44,222,484	6,633,372,600	96.10	90.60	57.57

The 30 samples including three biological replicates yielded a total of 1.48×10^9 150-bp paired-end reads and corresponding 221.72 Gbp of sequence dada. The Q20 and Q30 base ratios of these samples fell into ranges of 95.25%~96.83% and 88.99%~91.94% with corresponding average values of 96.07% and 90.62%, respectively. On the whole, various parameters of these sequencing data are relatively comparable and close to each other within Nip or Olr samples.

Table S4. Statistics of clean reads mapped to the Nip reference genome

Sample name	Total records	Unmapped reads	Ratio of unmapped reads (%)	Unique mapped reads	Ratio of unique mapped reads (%)	Multiple mapped reads	Ratio of multiple mapped reads (%)	Ratio of total mapped reads (%)
N01r1	51,263,834	3,898,627	7.90	44,396,670	90.02	1,025,041	2.08	92.10
N01r2	50,640,528	4,569,238	9.36	43,238,552	88.59	998,548	2.05	90.64
N01r3	51,220,321	4,901,279	9.93	43,423,983	87.99	1,027,224	2.08	90.07
N06r1	57,386,551	5,300,328	9.55	49,039,121	88.39	1,138,449	2.05	90.45
N06r2	46,342,202	3,617,345	8.11	39,999,538	89.67	992,517	2.22	91.89
N06r3	49,689,392	3,845,377	8.03	42,997,176	89.76	1,058,259	2.21	91.97
N12r1	94,137,582	8,356,252	9.51	75,125,938	85.47	4,411,030	5.02	90.49
N12r2	57,257,014	4,557,106	8.52	46,199,835	86.39	2,720,107	5.09	91.48
N12r3	47,593,778	4,947,945	10.99	38,249,740	84.97	1,817,137	4.04	89.01
N18r1	46,533,585	2,577,384	6.31	34,586,664	84.64	3,700,220	9.05	93.69
N18r2	46,544,012	2,840,519	7.37	31,287,083	81.22	4,391,454	11.40	92.63
N18r3	51,878,777	2,751,939	5.95	39,828,202	86.09	3,685,639	7.97	94.05
N24r1	57,440,260	3,135,094	6.44	39,984,926	82.15	5,550,908	11.40	93.56
N24r2	46,422,244	2,813,586	6.66	36,327,414	86.03	3,084,232	7.30	93.34
N24r3	51,852,062	3,416,717	7.01	43,547,184	89.39	1,753,921	3.60	92.99
O01r1	49,820,135	5,921,317	12.22	41,744,991	86.18	773,416	1.60	87.78
O01r2	51,552,369	6,471,045	12.91	42,812,689	85.45	821,754	1.64	87.09
O01r3	44,206,772	5,587,442	13.00	36,719,680	85.40	687,756	1.60	87.00
O06r1	50,485,071	5,905,224	12.15	41,549,895	85.49	1,149,405	2.36	87.85
O06r2	53,097,720	6,029,483	11.77	44,090,864	86.03	1,128,227	2.20	88.23
O06r3	47,837,993	5,001,235	10.95	39,174,836	85.80	1,480,815	3.24	89.05
O12r1	49,288,896	4,810,175	10.63	37,408,523	82.67	3,029,642	6.70	89.37
O12r2	51,364,344	5,362,195	11.31	39,013,395	82.30	3,025,558	6.38	88.69
O12r3	56,036,803	5,895,485	11.50	41,948,835	81.83	3,420,014	6.67	88.50
O18r1	59,499,383	5,446,324	10.20	43,712,232	81.88	4,228,332	7.92	89.80
O18r2	48,522,886	5,534,514	11.87	39,889,814	85.58	1,189,104	2.55	88.13
O18r3	45,326,716	4,401,021	10.14	37,778,238	87.02	1,235,097	2.84	89.86
O24r1	50,383,650	7,534,002	15.67	39,216,665	81.54	1,342,211	2.79	84.33
O24r2	70,917,394	9,355,690	14.55	51,177,949	79.57	3,787,251	5.89	85.45
O24r3	45,904,201	5,615,397	12.70	37,689,064	85.23	918,023	2.08	87.30

A total of 1.58×10^9 records were subsequently obtained after mapping all the paired-end reads to the Nip reference genome. The ratios of unique mapped reads and total mapped reads to the paired in sequencing reads range from 9.57%~90.02% and 84.33%~94.05%, with the average values of 85.42 ± 2.79% and 89.81 ± 2.52%, respectively. On the whole, various parameters of these mapping data are relatively comparable and close to each other within Nip or Olr samples.

Table S5. Statistics of differentially expressed genes

Comparison group	DEG set name	Up-regulated DEGs	Down-regulated DEGs	All DEGs
NS01_vs_OS01	S01	2522	4422	6944
NS06_vs_OS06	S06	3776	5807	9583
NS12_vs_OS12	S12	2975	4364	7339
NS18_vs_OS18	S18	3347	4174	7521
NS24_vs_OS24	S24	930	2055	2985

NS01_vs_OS01, NS06_vs_OS06, NS12_vs_OS12, NS18_vs_OS18 and NS24_vs_OS24 mean comparison groups of grain samples between Olr and Nip at 1DAF, 6 DAF, 12 DAF, 18 DAF and 24 DAF, respectively. Overall, the numbers of up-regulated DEGs are all lower than numbers of down-regulated DEGs in NS01_vs_OS01, NS06_vs_OS06, NS12_vs_OS12, NS18_vs_OS18 and NS24_vs_OS24 comparison groups, with a total of 13550 up-regulated and 20822 down-regulated DEGs of the five comparison groups. It partly suggests a trend of overall down-regulated expression of genes in Olr grains compared with Nip grains. Furthermore, in these five comparison groups, there are much more total, up-regulated and down-regulated DEGs in samples at early and middle stages (S01, S06, S12 and S18) than samples at later stage (S24) of grain development. It can be attributed to the different transcriptomic profiles of developing grains at different developmental stages, relatively active gene expressions in early and middle stages of developing grains but overall decreased gene expressions in nearly mature grains.

Table S6. Starch biosynthesis, degradation and related regulating genes in rice grains

Category	Gene accession number	Gene name	Encoding protein (enzyme)	Number of encoding protein (enzyme) in Figure 6	Catalytic reaction (reactions) / regulatory function (functions)
Starch biosynthesis pathway	LOC_Os04g33740	*GIF1*	Cell wall invertase 2	1	Sucrose → Fructose + Glucose
	LOC_Os07g42490	*SUS3*	Sucrose synthase 3	2	Sucrose → Fructose + UDP-glucose
	LOC_Os03g22120	*SUS4*	Sucrose synthase 4		
	LOC_Os01g71320	*OsHXK3*	Hexokinase 3	3	Glucose → G6P; Fructose → Fructose-6P
	LOC_Os08g02120	*OsFK1*	Fructokinase 1	4	Fructose → Fructose-6P
	LOC_Os03g56460	*PGI1*	Phosphoglucose isomerase 1	5	Fructose-6P → GLucose-6P
	LOC_Os10g11140	*OspPGM*	Plastidic phosphoglucomutase	6	Glucose-6P ⇌ Glucose-1P
	LOC_Os09g38030	*OsUgp1*	UDP-glucose pyrophosphorylase 1	7	UDP-glucose → Glucose-1P
Starch biosynthesis pathway	LOC_Os09g12660	*OsAGPS1*	ADP-glucose pyrophosphorylase small subunit 1	8	Glucose-1P → ADP-glucose
	LOC_Os08g25734	*OsAGPS2*	ADP-glucose pyrophosphorylase small subunit 2		
	LOC_Os05g50380	*OsAGPL1*	ADP-glucose pyrophosphorylase large subunit 1		
	LOC_Os01g44220	*OsAGPL2*	ADP-glucose pyrophosphorylase large subunit 2		
	LOC_Os06g04200	*Wx*	Granule-bound starch synthase	9	ADP-glucose → Amylose
	LOC_Os06g06560	*OsSSⅠ*	Starch synthase Ⅰ	10	ADP-glucose → Short chains of amylopectin
	LOC_Os06g12450	*OsSSⅡa*	Starch synthase Ⅱa	11	Short chains of amylopectin → Medium chains of amylopectin
	LOC_Os08g09230	*OsSSIIIa*	Starch synthase IIIa	12	Medium chains of amylopectin

					→ Long chains of amylopectin
Starch biosynthesis pathway	LOC_Os06g51084	*OsBE I*	Starch branching enzyme I	13	Branching chains of amylopectin
	LOC_Os02g32660	*OsBE IIb*	Starch branching enzyme II b		
	LOC_Os08g40930	*ISA1*	Starch-debranching enzyme, Isoamylase 1	14	Removing improper branches of amylopectin
	LOC_Os03g55090	*OsPho1*	Starch phosphorylase	15	Glucose-1P → Starch
Sucrose, glucose-6-phosphate and ADP-glucose transportor	LOC_Os03g07480	*OsSUT1*	Sucrose transporter 1	16	Transporting sucrose from extracellular space into cytosol
	LOC_Os12g44380	*OsSUT2*	Sucrose transporter 2		
	LOC_Os08g08840	*OsGPT1*	Glucose-6-phosphate translocator 1	17	Transporting Glucose-6P from cytoplasm into amyloplast
	LOC_Os02g10800	*OsBT1*	ADP-glucose transporter 1	18	Transporting ADP-glucose from cytoplasm into amyloplast
Regulation of starch biosynthesis	LOC_Os05g03040	*RSR1*	Rice AP2/EREBP family transcription factor	19	Negatively regulating starch biosynthesis
	LOC_Os02g49410	*OsNF-YB1*	Nuclear transcription factor Y subunit B-1	19	Positively regulating starch biosynthesis
	LOC_Os10g11580	*OsNF-YC12*	Nuclear transcription factor Y subunit C-12		
	LOC_Os04g35010	*bHLH144*	Helix-loop-helix DNA-binding domain containing protein		
	LOC_Os04g55230	*FLO2*	Tetratricopeptide repeat (TPR) domain containing protein		
	LOC_Os08g41940	*OsSPL16*	Squamosa promoter-binding-like protein 16		
	LOC_Os02g07430	*OsMADS29*	MADS box transcription factor 29		
Regulation of starch grain and	LOC_Os01g08420	*SSG4*	Amyloplast-localized protein containing DUF490	20	Regulating development of

amyloplast development	LOC_Os06g03990	*SSG6*	Protein homologous to aminotransferase		starch grain and amyloplast
	LOC_Os03g48170	*FLO6*	CBM48 domain-containing protein		
	LOC_Os08g01920	*FSE1*	Phospholipase-like protein homologous to phosphatidic acid-preferring phospholipase A1		
	LOC_Os10g32680	*FLO7*	A regulator protein of starch synthesis and amyloplast development with a domain of unknown function 1338 (DUF1338)		
Starch degradation pathway	LOC_Os02g52700	*RAmy1A*	Alpha-amylase 1A	21	Starch → α-maltose + α-glucose
	LOC_Os01g25510	*RAmy1C*	Alpha-amylase 1C		
	LOC_Os06g49970	*RAmy2A*	Alpha-amylase 2A		
	LOC_Os09g28400	*RAmy3A*	Alpha-amylase 3A		
	LOC_Os09g28420	*RAmy3B*	Alpha-amylase 3B		
	LOC_Os08g36910	*RAmy3D*	Alpha-amylase 3D		
	LOC_Os04g33040	*RAmy3E*	Alpha-amylase 3E		
	LOC_Os01g51754	*RAmy3F*	Alpha-amylase 3F		
	LOC_Os10g32810	*OsBamy1*	Beta-amylase 1	22	Starch → β-limit-dextrins + β-maltose
	LOC_Os03g04770	*OsBamy2*	Beta-amylase 2		
	LOC_Os01g13550	*OsBamy4*	Beta-amylase 4		

Table S7. Seed storage protein biosynthesis, trafficking, accumulation and related regulating genes in rice grains

Category	Gene accession number	Gene name	Encoding protein (enzyme)	Number of encoding proteins in Figure 7	Synthetized seed storage protein / regulatory function (functions)
Regulation of seed storage protein biosynthesis	*LOC_Os07g08420*	*RISBZ1*	Rice basic leucine zipper transcription factor	1	Positively regulating seed storage gene transcription
	LOC_Os02g15350	*RPBF*	Rice prolamin box binding factor		
Glutelin precursors biosynthesis	*LOC_Os01g55690*	*GluA1*	Glutelin precursor A1	2	Glutelin precursors
	LOC_Os10g26060	*GluA2*	Glutelin precursor A2		
	LOC_Os03g31360	*GluA3*	Glutelin precursor A3		
	LOC_Os02g15169	*GluB-1a*	Glutelin precursor B-1a		
	LOC_Os02g15178	*GluB-1b*	Glutelin precursor B-1b		
	LOC_Os02g15150	*GluB2*	Glutelin precursor B2		
	LOC_Os02g16830	*GluB4*	Glutelin precursor B4		
	LOC_Os02g16820	*GluB5*	Glutelin precursor B5		
	LOC_Os02g15070	*GluB6*	Glutelin precursor B6		
	LOC_Os02g14600	*GluB7*	Glutelin precursor B7		
	LOC_Os02g25640	*GluC-1*	Glutelin precursor C-1		
	LOC_Os02g15090	*GluD-1*	Glutelin precursor D-1		
Globulin biosynthesis	*LOC_Os05g41970*	*Globulin*	Globulin	3	Globulin
Prolamins biosynthesis	*LOC_Os03g55740*	*pro10.3*	Prolamin 10.3	4	Prolamins
	LOC_Os11g33000	*pro10.4*	Prolamin 10.4		
	LOC_Os07g10570	*pro13a.1*	Prolamin 13a.1		
	LOC_Os07g10580	*pro13a.2*	Prolamin 13a.2		
	LOC_Os12g16880	*pro13a.3*	Prolamin 13a.3		
	LOC_Os12g16890	*pro13a.4*	Prolamin 13a.4		
	LOC_Os07g11900	*pro13b.1*	Prolamin 13b.1		
	LOC_Os07g11910	*pro13b.2*	Prolamin 13b.2		
	LOC_Os07g11920	*pro13b.3*	Prolamin 13b.3		
	LOC_Os05g26350	*pro13b.8*	Prolamin 13b.8		
	LOC_Os06g31070	*pro16.2*	Prolamin 16.2		
Regulation of seed storage protein modification	*LOC_Os11g09280*	*OsPDIL1-1*	Protein disulphide isomerase-like enzyme	5	Regulating seed storage protein modification
	LOC_Os02g02410	*Bip1*	Endoplasmic reticulum chaperone		
Regulation of seed storage protein trafficking and accumulation	*LOC_Os03g11100*	*GOT1B*	Golgi transport	6	Regulating localization of prolamines and globulin mRNAs to the protein body ER (endoplasmic

					reticulum); regulating export of glutelin precursor and globulin from endoplasmic reticulum to Golgi apparatus
	LOC_Os01g23620	*OsSar1a*	Small GTPase OsSar1a	7	Regulating export of glutelin precursors and globulin from endoplasmic reticulum to Golgi apparatus
	LOC_Os12g37360	*OsSar1b*	Small GTPase OsSar1b		
	LOC_Os01g15010	*OsSar1c*	Small GTPase OsSar1c		
	LOC_Os12g43550	*OsRab5a*	Small GTPase OsRab5a	8	Regulating localization of glutelin precursors mRNAs to the cisternal ER (endoplasmic reticulum); regulating glutelin precursors and globulin trafficking from Golgi apparatus to PBⅡ (protein body Ⅱ) via DV (dense vesicle)
Regulation of seed storage protein trafficking and accumulation	*LOC_Os03g15650*	*OsVPS9a*	Guanine nucleotide exchange factor	9	Regulating glutelin precursors and globulin trafficking from Golgi apparatus to DV (dense vesicle)
	LOC_Os03g61950	*GPA3*	A regulator of post-Golgi vesicular traffic		
	LOC_Os06g43560	*GPA5*	A Rab5a effector	10	Regulating glutelin precursors and globulin trafficking from DV (dense vesicle) to PBⅡ (protein body Ⅱ)
	LOC_Os04g45470	*OsVPE1*	Vacuolar processing enzyme 1	11	Regulating shearing and maturation of glutelins from glutelin precursors

Table S8. Primers developed for vector construction

**Primers**	**Final vectors**	**Inserted vectors (Original vectors)**	**Sequence of forward and reverse primers (5′ - 3′)**
OE	p*Ubi*::*OsMADS1*	pC*Ubi*1390	F: CGCGGATCCATCAGGTAGCCAAACCACACCAC<br>R: GGACTAGTTGCCAATTAACTTGTTACCACATCC
MADS1 / *OsMADS1*^{Olr}-*eGFP*	pJIT163-P*35S*::*OsMADS1-eGFP* / pJIT163-P*35S*::*OsMADS1*^{Olr}-*eGFP*	pJIT163-P*35S*::*eGFP*	F: TGGAGAGGACAGCCCAAGCTTATGGGGAGGGGGAAGGTGG<br>R: GCCCTTGCTCACCATGGATCC-TATCCAGCCGGATGGGATG

Sequences marked by underline indicate the restriction site.

Table S9. Primers used for qRT-PCR analysis

Primers	Sequence of primers (5′ - 3′)
GluB-1a-F	GAACCAATGTGCAACACCAG
GluB-1a-R	GCCAAAGTCAGAGCCAAAAG
GluB-4-F	GGGTTGTGCCATGGATTTAC
GluB-4-R	TGGCGACCATAGCTTTCTCT
GluB7-F	GCGACCAGAAGGCTACAAAG
GluB7-R	TTGCTTGTTGATCGTTGCTC
GluA-1-F	GGTTGCAAGCATTTGAGCCA
GluA-1-R	GGCAACAACTGGCACTTCA
GluB2-F	CAAGCACAAACCCATGGCAT
GluB2-R	CAACAACCGATGCATCACCA
GluB-1b-F	CAAGACAAACGCTAACGCCTTC
GluB-1b-R	TCGATAATCCTGGGTAGTATTG
pro13a.2-F	CTACTACATTGCTCCGAGGAGC
pro13a.2-R	CGCATGATGATGCATGACTTT
pro13b.3-F	GCTTGCCGCAATGCTATACT
pro13b.3-R	CACAGCGCAGTTTGATGTTT
OsSar1d-F	GCTACGGCGATGGCTTCA
OsSar1d-R	CATGCTCTGAGTCTCTACCATGTTC
OsbZIP58-F	CAAGGGAGCCATCACCATC
OsbZIP58-R	CCTTTCTGCTTCTTGAGCGTCTA
RPBF-F	ACTACGCGCCTCTCATCACC
RPBF-R	TCACTCCACCACCACCTCCT
OsPDIL1-1-F	TTGCGTCTTCTGGTGACTTG
OsPDIL1-1-R	ACCAGGGCAAGAACATTCAG
